# Epidemiology, risk factors, and prevention strategies of HIV, HPV, and other sexually transmitted infections among cisgender and transgender youth: a narrative review

**DOI:** 10.3389/fpubh.2024.1342532

**Published:** 2024-03-07

**Authors:** Pierluigi Diana, Susanna Esposito

**Affiliations:** Pediatric Clinic, Pietro Barilla Children’s Hospital, Department of Medicine and Surgery, University Hospital of Parma, Parma, Italy

**Keywords:** adolescents, behavioral risk factors, gender minority youth, sexually transmitted infections, transgender, cisgender

## Abstract

Adolescents face an increased risk of contracting sexually transmitted infections (STIs) with alarming data especially concerning HIV. Limited data exists for teenagers regarding the influence of their gender identity (GI) and sexual orientation on the risk of STIs. This narrative review aims to analyse the available data to provide a comprehensive overview of STIs incidence and risk factors among adolescents, taking into account the unique circumstances related to various sexual orientations and GIs. Transgender and gender minority (TGM) youth experience more challenges accessing health services compared to cisgender youth. This is often attributed to non-inclusive health environments, barriers to obtaining medical gender affirmation, and an underestimation of sexual risk perception. Literature analysis has revealed that the majority of adolescents, both cisgender and TGM, have limited awareness regarding the risks associated with their sexual behaviors, the most common sexually transmitted diseases, and strategies for prevention, such as PrEP and HPV vaccination. Moreover, a significant portion of pediatricians possess limited knowledge and comfort in addressing various aspects of sexual health, particularly when it involves discussing topics such as sexual orientation, gender identity, and sexual behaviors with sexually active adolescents. This underscores the pressing need for enhanced education for pediatricians, specifically focusing on STIs diagnosis, prevention, and screening.

## 1 Introduction

Sexually transmitted infections (STIs) refer to a wide spectrum of bacterial, fungal, viral and protozoal infections that share a common pattern of transmission through sexual contact (1). It has been largely observed that adolescents are at increased risk for acquiring STIs. The World Health Organization (WHO) estimates that more than 1 million STIs affect people between the ages of 15 and 49 every day, and that number is constantly rising (2). Recent data from United States reported that of about 20 million new STIs every year, 50% of cases occur among youth aged 15–24 y.o (3). Similar data have been collected in Australia, reporting 25% of chlamydia infections diagnosed among people younger than 20 y.o (4). In Ireland, Davoren et al. reported that the incidence of STIs among adolescents was 225/100,000 person-years (5). Moreover, as reported by Viottini et al. the incidence of STIs in Italy has particularly increased in the population aged 15–24 y.o. across the last 10 years (6, 7). The literature has demonstrated that various risk factors, both biological and neuro-behavioral, make adolescents more susceptible to contracting sexually transmitted infections; however, such data are almost always reported in the pediatric population without taking into account different sexual orientations and expressions of gender identity (GI) (8–13). This narrative review aims to analyse the available data to provide a comprehensive overview of STIs incidence and risk factors among adolescents, taking into account the unique circumstances related to various sexual orientations and GIs.

## 2 Methods

This is a narrative review of literature on STIs among cisgender and transgender minority youth in pediatrics. Systematic searches were performed in Pubmed and Google Scholar up to September 2023. Language was restricted to English. Search terms included “STIs” OR “sexually transmitted infection” AND “cisgender” OR “transgender” OR “gender nonconforming” OR “non-Binary” in combination with “adolescents” OR “youth.” Original research studies, review articles, letters to the editor, cohort studies published between 2012 and 2023 were included. Relevant papers were scrutinized for additional sources not identified by the electronic search, thereby enriching the research findings through a snowballing approach. We excluded papers that did not focus specifically on adolescent cisgender and TGD populations. Data from earlier studies were taken into account if relevant to the scope of this review. All relevant articles were then evaluated, and pertinent articles were included in this review.

## 3 Risk factors

Main risks for increased STIs rates among adolescents regardless their sexual orientation and GI are summarized in Table 1.

**Table 1:** Risk factors for increased STIs rates among adolescents regardless of sexual orientation and GI.

Biological	Immature prefrontal cortex responsible for executive function
	Cervical ectopy and reduced cervical mucous in AFAB (higher risk for CT and HPV)
	Low circumcision rate in AMAB
	Lack of immunity of previous infection
Behavioral	Alcohol and drugs
	Low condom use
	Frequent oral sex
	Early sexual debut (<15 y.o.)
	Poor sexual education

STIs, sexually transmitted infections; GI, gender identity; AFAB, assigned female at birth; AMAB, assigned male at birth; CT, chlamydia trachomatis; HPV, human papilloma virus.

## 4 Epidemiology

Four treatable STIs, i.e., those due to *Trichomonas vaginalis* (42%), CT (34%), *Neisseria gonorrhoeae* (NG) (22%), and *Treponema pallidum* (2%), are mostly responsible for the 374 million new cases that have been reported globally in 2020 (2). According to additional WHO data, the Herpes Simplex Virus-2 (HSV-2) infection that causes genital herpes is currently affecting about 500 million adults, although the number of cases due to HSV-1 is now increasing (14). Additionally HPV, which is the main cause of anal cancer in males who have sex with men and cervical cancer in women, is present in more than 300 million women and 300 million men (6, 14). Moreover, there are resurgent STIs like *lymphogranuloma venereum* as well as new outbreaks of illnesses that can spread through sexual contact, including monkeypox, *Shigella sonnei, Neisseria meningitidis*, Ebola, and Zika (2). Recent data from USA enlightened the epidemiological context of STIs among the young population (12). Teenagers have been found to have the highest prevalence of CT and NG of any age group. In the population aged 15–19 years old, the CT rate infection has increased by 4.1% among girls and 15.3% among boys, while the NG rate infection has increased by 11.3%. In the same population and period, syphilis has increased by 24.5% (12).

There is lack of data regarding the HPV prevalence in the adolescent population (15). Markowitz et al. have estimated that 29% of adolescent girls aged 14–19 y.o. had HPV infection (16). Remarkably, the study has also highlighted the positive impact of the HPV vaccine against the infection rate in USA, reporting a 64% reduction in HPV 6, 11, 16 and 18 prevalence among adolescent girls. These findings fully agree with more recent studies. Ju et al. have reported a decrease of HPV 16 prevalence rate (35% in vs. 5%) in Swedish youth after HPV vaccine was offered to 10–12 years old girls through the school-based vaccination program (17). Similarly, in Spinner et al. findings HPV detection decreased from 35 to 6.7% among HPV vaccinated girls after 10 years from the vaccine introduction (18). Numbers may be different elsewhere according to HPV vaccination recommendations and coverage.

Sexually active youth also have a high risk of contracting human immunodeficiency virus (HIV) (8). As reported in data from USA, adolescents and young adults represented 4% of persons living with HIV infection in 2018, while teenagers made up 21% of all new HIV diagnoses (19, 20). According to Underwood et al. only 54% of sexually active high school adolescents reported condom use in their last sexual intercourse and this rate was even lower among homosexual adolescents (21). Those ones also reported a larger number of sexual partners in their lifetime compared to the heterosexual peers. Adolescents also have poorer viral suppression rates, which reduces their likelihood of maintaining good health and raises their risk of HIV transmission to others (22, 23).

## 5 Transgender and other gender minority youth

GI is the sense of being male, female, a combination of both, or neither (24). Individuals who identify as transgender (TGD) are those whose gender identification does not match the sex they were assigned at birth. Adolescence is a critical time for the development of GI, as young people navigate the completion of developmental tasks such as forming an identity, developing relationships, and exploring sexuality (8).

It has been observed that transgender and gender minority youth (TGMY) experience unique risks that can lead to increased rates of STIs and sexual risk behaviors (25). As described by Phillips et al. one of the factors that must be taken into account could be the role of minority stress, which is the excessive stress experienced by a marginalized community as a result of proximal (e.g., perceived stigma) and distal (e.g., systemic stigmatization) elements (26). Together with the gender affirmation framework, which relates to the way social affirmation/non-affirmation of one’s perceived gender may influence behavioral risk factors, this has been related to potentially dangerous experiences for TGMY (27). Adolescents who experience non-inclusive background at school as well as non-inclusive families might be more likely to incur into loss of health services, engagement in sex work, weak social support networks, all factors associated with HIV risk (28–32). Reisner et al. have reported that 31% of transgender youth had been engaged in any condomless receptive or insertive intercourse (vaginal and/or anal) within the last 6 months (25). Moreover, 33% had ever diagnosed with an STI in their lifetime and 55% of transgender girls had been diagnosed with syphilis (25).

Among STIs, HIV represents a critical situation. Data available from a recent USA surveillance report estimated that 21% of all new HIV diagnoses in 2019 and 45% of all undiagnosed HIV cases affect young people aged between 13 and 24 years old (19, 33). We still have lack of data among TGMY; however, it has been estimated an HIV prevalence of 9.2% for all TGD persons in USA. In view of this, we can expect that in the same way TGMY are at disproportionate risk of being infected (34). Fisher et al. have reported that 58% of TGMY aged 14–17 years had had at least one lifetime sexual partner and that the majority of adolescents believed extremely unlikely to be infected with HIV (35). Nevertheless, Reisner et al. have showed a high proportion of TGD boys that engaged into condomless receptive anal sex (41% against 2.4% among TGD girls), which is the most efficient HIV transmission risk behavior (25). Sevelius et al. highlighted that the 52.9% of TGMY had never tested for HIV and the 66% had never tested for any STDs (27). Compared to TGD peers, condom use at last sex was more likely among cisgender adolescents (36).

According to research, TGMY may encounter the same obstacles to HIV prevention programs catered to their needs as TGD adults. These obstacles include a lack of TGD-friendly and competent clinicians and uncomfortable talking about TGD-specific health issues with medical doctors (37–40). In according with these findings, in the Fisher et al. study population nearly half of the participants said their medical provider was ignorant of their GI and expressed worry that disclosure would lead to rejection; moreover, younger participants showed higher levels of gender-related stigma (35). Compared to cisgender peers, TGD adolescents seem to receive a pourer sex education. As observed by Bloom et al. sexual education provided among American schools focuses predominantly on pregnancy prevention and cis-heterosexual intercourses (41). Literature showed that medical gender affirmation may increase awareness of HIV and STIs prevention; after affirming their GI, young individuals may be more likely to engage in self-care or health-promoting actions (27).

Table 2 reports main risks for increased STIs rates among TGMY.

**Table 2:** Risk factors for increased STIs rates among TGMY.

Minority stress	Perceived stigma	Non-inclusive school environment Non-inclusive health services Family rejection Weak social support networks Delayed medical gender affirmation
	Systemic stigmatization	

STIs, sexually transmitted infections; TGMY, transgender and gender minority youth.

## 6 Prevention of HIV and HPV

To contain the continuous increase in STIs among adolescents, it is essential to instill awareness of the importance of prevention.

Concerning HIV, pre-exposure prophylaxis (PrEP) has been found to be effective in reducing the risk of HIV transmission by >90% (42, 43). Recent research has revealed that TGMY have poor PrEP awareness and very low uptake despite the medication’s well-known effectiveness (44). In accordance with this, Horvath et al. have reported that almost 44% of TGMY have never heard about PrEP and only 0.5% were currently using PrEP (45). The majority of individuals did not want to use PrEP because they believed their personal level of HIV risk to be low (45). Similar data were collected by other authors among TGD adults (27, 46–48).

As underlined by the American Academy of Pediatrics (AAP) in 2022, the majority of pediatricians still experiment lack of knowledge, personal discomfort discussing sexual issues with adolescents and insufficient training in how to treat and prevent STIs and HIV. However, pediatricians should play a key role in preventing and controlling HIV infection (49). Therefore, it has been recommended routine HIV screening for all youth 15 years or older at least once in health care setting; then those with increased risk (i.e., sexually active adolescents) should receive a screening every year and those with high risk (i.e., male youth who report same sex contact, substance abusers, TGMY, partners of HIV-infected ones or of drug users) should be rescreened every 3–6 months (49). As part of an all-encompassing preventative plan that also includes other prevention measures (such as safer sex behaviors and use of barrier protection) to lower the risk of STIs, the AAP highlighted that HIV PrEP should be made available to all youth at risk for HIV acquisition. For young people who may have been exposed to HIV during a period of high-risk sexual activity, unsafe needle use, or sexual assault, HIV post-exposure prophylaxis (PEP) along with antiretroviral medication should be taken into consideration (49).

With Resolution of the BoD No. 15 of April 26, 2023, the Italian Medicines Agency has admitted the reimbursability of the association Emtricitabine/Tenofovir Disoproxil for PrEP in order to reduce the risk of sexually transmitted HIV infection in adults and adolescents at high risk (50). As PrEP, it is recommended to administrate Emtricitabine/Tenofovir Disoproxil in adolescents aged 12 years and older (51).

HPV vaccine is recommended for preventing precancerous lesions, malignancies of the cervix, vulva, vagina, penis, and anus, as well as genital *condyloma acuminata*, which are caused by HPV subtypes (52). Given before the start of sexual activity, the HPV vaccination is most effective. According to data, 11% of girls and 16% of males had their first sexual experience by the age of 15 years, with the first sexual encounter occurring on average at 17 y.o (53). It is crucial to be immunized before the age of 13 years, as 2% of female and 5% of male adolescents are thought to have had their sexual debut before turning 13 (54). As summarized by Bednarczyk et al., evidence for prevention of high-grade pre-cancers and cervical cancer is strong, with several clinical trials documenting HPV vaccines efficacy against CIN2+ of 95 to 98 and 100% against high grade vaginal and vulvar lesions (55–57).

Along with promotion of the available vaccinations, it is crucial that pediatricians provide inclusive sex education, with an eye on both cisgender and TGMY, which can result in delayed sex debut, increased condom use and reduced sexual risk behaviors (58, 59).

## 7 Conclusion

STIs represent a common cause of disease among all adolescents. Literature analysis showed increasing rates of STIs overall in this population, with alarming data particularly related to HPV in those who did not receive the vaccine, and HIV. Our literature review has highlighted a significant lack of data regarding the sexual health of cisgender and, even more so, transgender adolescents in European and Italian studies; for this reason, many of the presented data come from American studies. However, it is important to emphasize how the specific situation of transgender and cisgender youth can vary significantly across countries, even among Western countries. Moreover, the majority of pediatricians have limited knowledge and comfort on many items regarding sexual health, especially when it comes to discuss sexual orientation, GI and sexual behaviors with sexually active adolescents. Compared with cisgender youth, TGMY experiment worse access to health services due to a non-inclusive health environment, difficult access to medical gender affirmation and underestimated sexual risk perception. Specific training should be provided to pediatricians related to TGMY care, including how to discuss about their sexual identity, how to share information about STIs screening, particularly about HIV testing and PrEP. Both cisgender and TGD youth still have poor PrEP awareness, which leads to lack of access to the medication even where it is available for free, and their HIV personal risk is perceived as low by the majority of them. In addition, a strong communication about the importance of some vaccines against STIs should be done with all adolescent patients. Finally, due to the limited data, further studies are urgently needed on the epidemiology of STIs among patients aged under 18 years with non-heterosexual orientation and non-cisgender identity.

## Author contributions

PD: Conceptualization, Data curation, Investigation, Writing – original draft. SE: Project administration, Supervision, Validation, Writing – review & editing.
